# Extracutaneous features and complications of the Ehlers-Danlos syndromes: A systematic review

**DOI:** 10.3389/fmed.2023.1053466

**Published:** 2023-01-23

**Authors:** Brent J. Doolan, Mark E. Lavallee, Ingrid Hausser, Jane R. Schubart, F. Michael Pope, Suranjith L. Seneviratne, Ingrid M. Winship, Nigel P. Burrows

**Affiliations:** ^1^School of Basic and Medical Biosciences, St. John’s Institute of Dermatology, King’s College London, London, United Kingdom; ^2^Department of Orthopedics, University of Pittsburgh Medical Center of Central PA, Pittsburgh, PA, United States; ^3^Institute of Pathology, Heidelberg University Hospital, Heidelberg, Germany; ^4^Department of Surgery, Penn State College of Medicine, Hershey, PA, United States; ^5^Department of Dermatology, Chelsea and Westminster Hospital NHS Foundation Trust (West Middlesex University Hospital), London, United Kingdom; ^6^Institute of Immunity and Transplantation, Royal Free Hospital and University College London, London, United Kingdom; ^7^Nawaloka Hospital Research and Education Foundation, Nawaloka Hospitals, Colombo, Sri Lanka; ^8^Department of Genetic Medicine, The Royal Melbourne Hospital, Melbourne, VIC, Australia; ^9^Department of Medicine, The University of Melbourne, Melbourne, VIC, Australia; ^10^Department of Dermatology, Cambridge University Hospitals NHS Foundation Trust, Cambridge, United Kingdom

**Keywords:** Ehlers-Danlos syndrome, heritable connective tissue disorders (HCTD), extracutaneous, complications, joint hypermobility

## Abstract

**Introduction:**

The Ehlers-Danlos syndromes (EDS) comprise a group of inherited connective tissue disorders presenting with variable fragility to skin, soft tissue, and certain internal organs, which can cause significant complications, particularly arterial rupture, bowel perforation and joint difficulties. Currently, there are 14 proposed subtypes of EDS, with all except one subtype (hypermobile EDS) having an identified genetic etiology. An understanding of the extracutaneous features and complications within each subtype is key to maximizing clinical care and reducing the risk of further complications.

**Methods:**

A systematic review of EDS-related extracutaneous features and complications was undertaken.

**Results:**

We identified 839 EDS cases that met the inclusion criteria. We noted a high prevalence of joint hypermobility amongst kyphoscoliotic (39/39, 100%), spondylodysplastic (24/25, 96.0%), and hypermobile (153/160, 95.6%) EDS subtypes. The most common musculoskeletal complications were decreased bone density (39/43, 90.7%), joint pain (217/270, 80.4%), and hypotonia/weakness (79/140, 56.4%). Vascular EDS presented with cerebrovascular events (25/153, 16.3%), aneurysm (77/245, 31.4%), arterial dissection/rupture (89/250, 35.5%), and pneumothorax/hemothorax. Chronic pain was the most common miscellaneous complication, disproportionately affecting hypermobile EDS patients (139/157, 88.5%). Hypermobile EDS cases also presented with chronic fatigue (61/63, 96.8%) and gastrointestinal complications (57/63, 90.5%). Neuropsychiatric complications were noted in almost all subtypes.

**Discussion:**

Understanding the extracutaneous features and complications of each EDS subtype may help diagnose and treat EDS prior to the development of substantial comorbidities and/or additional complications.

**Systematic review registration:**

https://www.crd.york.ac.uk/prospero/display_record.php?ID=CRD42022308151, identifier CRD42022308151.

## Introduction

The Ehlers-Danlos syndromes (EDS) are a group of rare, inherited connective tissue disorders caused by pathogenic variants in genes responsible for matrix organization including those for collagen synthesis and processing, and glycosaminoglycans metabolism (1). Presently, there are 13 distinct clinical EDS subtypes, as outlined in the 2017 revised EDS international classification system from the International EDS Consortium (see Supplementary Table 1 for subtypes and the minor/major criteria for diagnosis) (2). Since this classification, a further gene variant for EDS has been identified (AE-binding protein 1; *AEBP1*), leading to a 14th subtype (classical-like type 2 EDS; clEDS2) and expanding the list of EDS-associated genes (3).

EDS varies according to specific subtype, but most presentations include some degree of skin hyperextensibility, joint hypermobility and/or fragility of skin, soft tissues and certain internal organs (bowel perforation, hernias, and arterial rupture) (4). The true prevalence and incidence of EDS in the general population is difficult to ascertain, as people with milder forms of joint and skin manifestations may not seek medical care. Amongst those presenting with EDS, hypermobile EDS (hEDS) is the most frequent, with classical (cEDS), vascular (vEDS), and hEDS collectively accounting for > 90% of patients, with some subtypes [dermatosparaxis EDS (dEDS) and clEDS2] limited to a handful of family pedigrees globally (5).

Variants in the genes responsible for normal matrix organization with collagen and extracellular matrix (ECM) function have an immense impact on both the skin and internal organs, as well as the integrity of the vasculature (4). Genetic defects affecting the biosynthesis and/or structure of fibrillar procollagens cause classical (defect in type V/I collagens, OMIM 130000), vascular (defect in type III/I collagens, OMIM 130050), arthrochalasia (aEDS; defect in type I collagen, OMIM 130060), and cardiac valvular (cvEDS; defect in type I collagen, OMIM 225320) EDS subtypes (1). Dermatosparaxis EDS (dEDS; enzyme defect in procollagen N-peptidase, OMIM 225410) results in poorly structured and loosely arranged collagen fibrils in the ECM. Similarly, classical-like EDS variants (clEDS; defect in tenascin-X ECM glycoprotein, OMIM 606408) result in a loss of anti-adhesive interactions with collagen in the ECM (1). Defects in the ECM bridging molecules have been implicated in clEDS2 [deficiency in aortic carboxypeptidase-like protein, OMIM 618000] and myopathic EDS (mEDS; defect in type 12 collagen, OMIM 616471) subtypes (4).

In kyphoscoliotic EDS (kEDS; OMIM 225400), deficiency in lysyl hydroxylase (*PLOD1*) or FKBP22 protein (*FKBP14*) cause the destabilization of intermolecular collagen crosslinking and folding (6). Subtypes such as musculocontractural EDS [mcEDS; deficiency in dermatan-4-O-sulfotransferase (*CHST14*) or dermatan sulfate epimerase (*DSE*), OMIM 601776] and spondylodysplastic EDS [spEDS; deficiencies in galactosyl-transferase I (*B4GALT7*) or galactosyl-transferase II (*B3GALT6*), OMIM 130070/615349] cause specific disorders of glycosaminoglycan biosynthesis (2), whilst subtypes with more localised phenotypic features such as brittle cornea syndrome (BCS, deficiency in ZNF469 or PRDM5 proteins, OMIM 229200) and periodontal EDS (pEDS, deficiency in C1r/C1s components, OMIM 130080/617174) have been associated in dysregulation of the intracellular processes within the ECM and the complement pathway respectively (7, 8).

The candidate genes that are responsible for the various EDS subtypes also cause development of extracutaneous manifestations. Understanding these specific changes is crucial, as it allows for accurate clinical subclassification of these manifestations, can help guide a clinician to a clinical diagnosis of EDS and can provide additional evidence to support the use of next generation sequencing technologies for a molecular diagnosis. Chronic deficits in these downstream pathways may also give rise to substantial long-term complications, particularly within the musculoskeletal, vascular, ocular, gastrointestinal, respiratory, and psychiatric systems. Some of these complications are potentially fatal (i.e., arterial rupture, bowel perforation, and pneumothorax) whilst other complications can cause a significant health burden for affected individuals. It must also be stressed that each EDS subtype is unique and is associated with an entirely separate clinical presentation and set of possible complications.

Thus, an assessment of the extracutaneous manifestations of EDS, as well as the complications associated with each EDS subtype is required to improve the clinical diagnosis of EDS and to prevent EDS-associated complications. Owing to the rarity of this complex group of genetic disorders, the paucity of evidence outlining the extracutaneous features and complications of EDS and the introduction of an additional subtype since the 2017 re-classification of EDS, a systematic review was undertaken.

## Materials and methods

A systematic search of the literature was conducted to identify published data pertaining to EDS. The databases included PubMed and Web of Science. Database searches were performed from the date of publication of the 2017 International Classification of EDS (March 2017) until 15 January 2022. Search strings were used to examine electronic databases for relevant references (see Supplementary Table 2). Search results were screened initially by title and abstract. Reference lists of included studies were screened for additional studies. Clinical findings for each EDS subtype were defined as per the 2017 International Classification of EDS (2). Articles were excluded if they did not contain a formal diagnosis of EDS as per the subtype classification. Only original, published cases in humans were included. Animal models were not included as they did not accurately predict human responses and complications. This study followed PRISMA guidelines (9).

Data extraction and aggregation were performed by a single investigator (BD), with uncertainties resolved through discussion with the principal investigator (NB). After identifying potential articles, the EDS Skin Working Group reviewed the papers to establish a consensus on inclusion. For each case, demographics, variant, extracutaneous features, Beighton scores, and complications were included. Joint pain was defined as pain associated with one or more joints, whilst chronic pain was defined as pain in one or more anatomic regions that persists or recurs for longer than 3 months and is associated with significant emotional distress or significant functional disability and that cannot be better explained by another chronic pain condition (10). For complications involving body systems (i.e., cardiovascular, gastrointestinal, urological, ocular, and neuropsychiatric) a broad group of findings were collected, some of which could be limited to, or have no significance in their association to EDS (refer to the footnote in Table 2 for a comprehensive list of findings for each system). Complications such as aneurysms, arterial dissection, and rupture events were excluded from the overall cardiovascular events and collected individually due to their clinical significance.

**TABLE 1:** Demographics, inheritance, and extracutaneous features of patients with Ehlers-Danlos syndrome.

Characteristics	Total	EDS subtype													
	All cases (*n* = 839)	cEDS (*n* = 171)	hEDS (*n* = 172)	vEDS (*n* = 305)	clEDS (*n* = 25)	aEDS (*n* = 13)	dEDS (*n* = 4)	kEDS (*n* = 40)	BCS (*n* = 11)	spEDS (*n* = 25)	mcEDS (*n* = 17)	mEDS (*n* = 5)	pEDS (*n* = 37)	cvEDS (*n* = 1)	clEDS2 (*n* = 13)
**Sex, n (%)**															
Male	285 (42.3)	36 (35.3)	5 (6.7)	159 (52.1)	6 (24.0)	7 (53.8)	1 (25.0)	14 (35.0)	3 (27.3)	15 (60.0)	8 (47.1)	3 (60.0)	21 (56.8)	0 (0.0)	7 (53.9)
Female	388 (57.7)	66 (64.7)	70 (93.3)	146 (47.9)	19 (76.0)	6 (46.2)	3 (75.0)	26 (65.0)	8 (72.7)	10 (40.0)	9 (52.9)	2 (40.0)	16 (43.2)	1 (100.0)	6 (46.1)
**Age at presentation (years)**															
Mean ± SD or Median (Q1, Q3)*	30.6 ± 17.9	30.5 ± 20.7	41.4 ± 14.2	29.8 ± 15.7	36.4 ± 15.2	22.0 (16, 29)	51.5 (40, 58)	12.2 ± 11.6	6.0 (4, 24)	13.3 ± 10.7	14.0 ± 7.5	7.0 (4,8)	26.5 ± 20.9	24.0	38.0 (24, 41)
**Genetic inheritance, n (%)**															
Positive family history	459 (60.0)	106 (66.2)	140 (77.9)	152 (51.7)	7 (25.0)	4 (30.8)	3 (75.0)	9 (23.7)	10 (90.9)	9 (36.0)	2 (66.7)	0 (0.0)	36 (97.3)	1 (100.0)	5 (55.6)
Autosomal dominant	456 (76.8)	99 (97.1)	−	299 (99.0)	0 (0.0)	12 (100.0)	4 (100.0)	0 (0.0)	0 (0.0)	0 (0.0)	0 (0.0)	4 (80.0)	37 (100.0)	0 (0.0)	1 (7.7)
*De novo*	73 (16.0)	28 (28.3)	−	44 (14.7)	0 (0.0)	1 (8.3)	0 (0.0)	0 (0.0)	0 (0.0)	0 (0.0)	0 (0.0)	0 (0.0)	0 (0.0)	0 (0.0)	0 (0.0)
Autosomal recessive	138 (23.2)	3 (2.9)	−	3 (1.0)	25 (100.0)	0 (0.0)	0 (0.0)	40 (100.0)	11 (100.0)	25 (100.0)	17 (100.0)	1 (20.0)	0 (0.0)	1 (100.0)	12 (92.3)
**Extracutaneous features**															
Joint hypermobility	534 (74.5)	124 (77.0)	153 (95.6)	139 (52.3)	3 (100.0)	13 (100.0)	3 (100.0)	39 (100.0)	6 (75.0)	24 (96.0)	3 (100.0)	4 (80.0)	9 (52.9)	1 (100.0)	13 (100.0)
Beighton score (≥ 5)	286 (73.5)	48 (62.3)	152 (88.9)	20 (36.4)	19 (82.3)	−	2 (66.7)	26 (96.3)	2 (50.0)	4 (100.0)	−	2 (100.0)	1 (8.3)	1 (100.0)	9 (90.0)
Pes planus	140 (74.1)	48 (87.3)	1 (100.0)	22 (48.9)	17 (77.3)	11 (84.6)	−	13 (56.5)	4 (100.0)	7 (100.0)	2 (100.0)	3 (60.0)	1 (100.0)	1 (100.0)	10 (100.0)
Craniofacial anomalies^†^	217 (57.9)	21 (32.8)	−	139 (57.0)	−	12 (92.3)	−	8 (72.7)	−	22 (95.7)	4 (100.0)	3 (60.0)	3 (100.0)	−	5 (62.5)
Scoliosis	152 (74.5)	45 (76.3)	0 (0.0)	19 (55.9)	2 (100.0)	8 (66.7)	0 (0.0)	35 (89.7)	2 (100.0)	15 (93.8)	13 (81.3)	1 (100.0)	5 (41.7)	1 (100.0)	6 (75.0)
Kyphosis	86 (61.9)	12 (27.3)	−	4 (80.0)	1 (100.0)	5 (41.7)	0 (0.0)	30 (85.7)	1 (100.0)	12 (100.0)	14 (100.0)	−	4 (36.4)	1 (100.0)	2 (100.0)
Blue/gray sclera	143 (58.6)	35 (56.5)	47 (48.0)	23 (70.0)	1 (100.0)	5 (38.5)	1 (100.0)	7 (87.5)	8 (100.0)	9 (100.0)	3 (100.0)	2 (40.0)	−	1 (100.0)	1 (50.0)
Chest wall abnormality	43 (39.8)	15 (27.8)	−	6 (100.0)	−	1 (8.3)	−	11 (44.0)	−	2 (100.0)	2 (100.0)	2 (100.0)	2 (100.0)	−	2 (66.7)
Arachnodactyly	18 (39.1)	3 (10.3)	−	1 (100.0)	−	−	−	2 (100.0)	5 (100.0)	4 (100.0)	1 (33.3)	−	−	−	2 (100.0)
Gingival inflammation	40 (38.1)	12 (23.1)	1 (100.0)	18 (41.9)	−	−	−	−	−	−	−	−	9 (100.0)	−	−
Thin cornea	9 (100.0)	−	−	−	−	−	−	1 (100.0)	8 (100.0)	−	−	−	−	−	−

cEDS, classical; clEDS, classical-like; vEDS, vascular; hEDS, hypermobile; aEDS, arthrochalasia; dEDS, dermatosparaxis; kEDS, kyphoscoliotic; BCS, brittle cornea syndrome; spEDS, spondylo-dysplastic; mcEDS, musculocontractural; mEDS, myopathic; pEDS, periodontal; cvEDS, cardiac valvular; clEDS2, classical-like 2; SD, standard deviation; Q1, first quartile; Q3, 3rd quartile; “-”, data not reported; N/A, not available.

*Mean or median chosen based on distribution of data range.

^†^Prominent high forehead, malar hypoplasia, depressed nasal bridges, low set and prominent ears, minimal prognathism, and short philtrum, narrow mouth and/or short face.

**TABLE 2:** Complications associated with various sub-types of the Ehlers-Danlos syndromes.

Characteristics, n (%)	Total	EDS subtype													
	All cases (*n* = 839)	cEDS (*n* = 171)	hEDS (*n* = 172)	vEDS (*n* = 305)	clEDS (*n* = 25)	aEDS (*n* = 13)	dEDS (*n* = 4)	kEDS (*n* = 40)	BCS (*n* = 11)	spEDS (*n* = 25)	mcEDS (*n* = 17)	mEDS (*n* = 5)	pEDS (*n* = 37)	cvEDS (*n* = 1)	clEDS2 (*n* = 13)
**Musculoskeletal complications**															
Sprain	55 (51.4)	27 (57.4)	12 (92.3)	11 (27.5)	1 (100.0)	−	−	2 (100.0)	−	0 (0.0)	−	−	−	0 (0.0)	2 (100.0)
Subluxation/dislocation	243 (49.5)	86 (62.8)	46 (43.4)	35 (23.3)	19 (79.2)	12 (100.0)	1 (100.0)	17 (60.7)	0 (0.0)	14 (93.3)	1 (100.0)	2 (40.0)	−	0 (0.0)	10 (100.0)
Muscle/tendon rupture	28 (11.1)	2 (6.5)	−	24 (11.0)	−	−	−	−	−	−	−	−	−	1 (100.0)	1 (100.0)
Joint pain	217 (80.4)	23 (46.0)	161 (94.7)	2 (40.0)	13 (59.1)	−	3 (100.0)	−	−	2 (100.0)	1 (100.0)	1 (100.0)	8 (61.5)	1 (100.0)	2 (100.0)
Osteoarthritis	8 (17.4)	8 (17.8)	0 (0.0)	0 (0.0)	−	−	−	−	−	−	−	−	−	−	−
Decreased bone density	39 (90.7)	6 (85.7)	−	3 (100.0)	2 (100.0)	−	−	5 (71.4)	2 (100.0)	15 (100.0)	1 (100.0)	−	−	0 (0.0)	5 (100.0)
Hypotonia/Weakness	79 (56.4)	6 (12.5)		5 (100.0)	15 (68.2)	7 (63.6)	−	23 (100.0)	−	13 (76.5)	2 (66.7)	4 (80.0)	−	−	4 (66.7)
Hernias	114 (26.4)	42 (31.8)	14 (12.8)	33 (21.3)	1 (100.0)	6 (50.0)	1 (100.0)	2 (66.7)	1 (100.0)	1 (100.0)	1 (100.0)	4 (80.0)	3 (100.0)	−	5 (62.5)
**Vascular complications**															
Cerebrovascular*	38 (23.2)	5 (100.0)	−	25 (16.3)	−	−	−	4 (100.0)	−	2 (100.0)	−	−	1 (100.0)	−	−
Carotid cavernous fistulae	7 (4.2)	−	−	7 (28.0)	−	−	−	−	−	−	−	−	−	−	−
Carotid event	11 (6.7)	−	−	11 (44.0)	−	−	−	−	−	−	−	−	−	−	−
Intracranial aneurysm	5 (3.0)	1 (20.0)	−	4 (16.0)	−	−	−	−	−	−	−	−	−	−	−
Intracranial event	15 (9.1)	4 (80.0)	−	7 (28.0)	−	−	−	4 (100.0)	−	2 (100.0)	−		1 (100.0)	−	−
Cardiovascular^†^	279 (54.7)	45 (63.4)	108 (63.5)	78 (44.6)	7 (29.2)	2 (18.2)	3 (100.0)	12 (66.7)	2 (33.3)	10 (71.4)	1 (50.0)	−	3 (75.0)	1 (100.0)	7 (63.6)
Extracranial aneurysm	89 (33.2)	5 (55.6)	0 (0.0)	77 (31.4)	−	−	−	2 (66.7)	−	−	−	−	4 (50.0)	−	1 (50.0)
Arterial dissection/Rupture	105 (38.3)	6 (54.5)	0 (0.0)	89 (35.5)	1 (100.0)	−	1 (100.0)	4 (100.0)	−	−	−	−	3 (100.0)	−	1 (50.0)
Cardiovascular dysautonomia	77 (65.8)	14 (29.8)	56 (88.9)	2 (100.0)	−	1 (100.0)	2 (100.0)	−	−	−	−	−	−	−	2 (100.0)
**Miscellaneous complications**															
Chronic pain	191 (73.7)	22 (43.1)	139 (88.5)	16 (43.2)	2 (100.0)	−	3 (100.0)	2 (100.0)	−	2 (100.0)	1 (100.0)	1 (100.0)	−	−	3 (100.0)
Chronic fatigue	104 (72.2)	20 (39.2)	61 (96.8)	2 (66.7)	15 (71.4)	−	2 (100.0)	−	−	−	−	1 (100.0)	−	−	3 (100.0)
Pneumothorax/ Hemothorax	42 (22.2)	1 (100.0)	−	40 (21.4)	−	−	−	−	−	1 (100.0)	−	−	−	−	−
Gastrointestinal^‡^	137 (67.8)	34 (66.7)	57 (90.5)	28 (53.8)	7 (29.2)	−	−	2 (100.0)	−	2 (100.0)	1 (100.0)	−	1 (100.0)	−	5 (83.3)
Bowel perforation	84 (30.4)	1 (100.0)	−	75 (30.0)	5 (22.7)	−	−	1 (100.0)	−	−	−	−	1 (100.0)	−	1 (100.0)
Urological^§^	98 (28.5)	16 (33.3)	59 (37.1)	12 (9.7)	−	−	1 (100.0)	3 (100.0)	−	2 (100.0)	2 (100.0)	−	1 (100.0)	−	2 (50.0)
Hearing loss	17 (54.8)	2 (66.7)	−	1 (100.0)	−	−	−	9 (64.3)	4 (44.4)	1 (25.0)	−	−	−	−	−
Ocular^¶^	141 (70.1)	44 (65.7)	46 (74.2)	7 (87.5)	3 (100.0)	4 (33.3)	1 (100.0)	8 (66.7)	7 (100.0)	9 (75.0)	3 (100.0)	1 (20.0)	−	1 (100.0)	7 (87.5)
Neuropsychiatric^#^	105 (73.4)	43 (75.4)	1 (100.0)	13 (92.9)	2 (66.7)	4 (33.3)	3 (100.0)	11 (100.0)	−	13 (59.1)	1 (33.3)	5 (100.0)	7 (100.0)	0 (0.0)	2 (50.0)

cEDS, classical; clEDS, classical-like; vEDS, vascular; hEDS, hypermobile; aEDS, arthrochalasia; dEDS, dermatosparaxis; kEDS, kyphoscoliotic; BCS, brittle cornea syndrome; spEDS, spondylo-dysplastic; mcEDS, musculocontractural; mEDS, myopathic; pEDS, periodontal; cvEDS, cardiac valvular; clEDS2, classical-like 2; SD, standard deviation; Q1, first quartile; Q3, 3rd quartile; “-”, data not reported; N/A, not available.

*Carotid events include carotid dissection/infarction, whilst intracranial events include intracranial hemorrhage and/or infarction. Cerebrovascular events were not mutually exclusive.

^†^Early onset varicose veins, valvular issues, arterial tortuosity, fistulae, murmurs, and bundle-branch blocks.

^‡^Constipation, dierrhoea, irritable bowel syndrome, reflux, and/or hepatitis.

^§^ Urinary incontinence, bladder cysts, bladder rupture, and/or uterine rupture.

^¶^ Myopia, astigmatism, keratoconus, cornea perforation, lens dislocation, glaucoma, and/or scleral ectasia.

^#^Delayed motor and cognitive development, migraines, tinnitus, dizziness, depression, anxiety, and/or attention-deficit disorder.

The mean and standard deviation (SD) is presented for parametric variables, and the median and interquartile range is presented for non-parametric variables. Categorical variables are presented as frequencies together with proportions. Categorical data were compared with Chi-square tests. Statistical significance was defined as *p* < 0.05.

## Results

Our search strategy identified 139 papers containing 839 EDS cases, which met the inclusion criteria (Supplementary Figure 1). There was a female-to-male ratio of 1.36:1 (*p* < 0.01), though higher ratios were noted for hEDS (11.8:1, *p* < 0.001) and clEDS (2.6:1, *p* < 0.001) subtypes. Genetic inheritance revealed predominantly autosomal dominant transmission, with some *de novo* involvement, reflecting the large cohort of cEDS and vEDS cases. A positive family history was present in 60.0% (459/795) of cases, with a higher prevalence noted in rarer autosomal recessive EDS subtypes, capturing family pedigree case studies. Demographics, genetic analysis, and extra-cutaneous features were collected (Table 1), as well as EDS-associated complications (Table 2).

### Facial features

Craniofacial abnormalities (i.e., prominent high forehead, malar hypoplasia, depressed nasal bridges, low-set and prominent ears, prognathism, short philtrum, and/or short face) were variably observed in spEDS (22/23, 95.7%), aEDS (12/13, 92.3%), and to a lesser extent in vEDS (139/244, 57.0%) subtypes. An expected high prevalence of blue/gray sclera (8/8, 100%) and thin cornea (8/8, 100%) were noted in BCS. Similarly, gingival inflammation was a prominent feature amongst pEDS cases (9/9, 100%).

### Musculoskeletal complications

Overall, we noted a high prevalence of joint hypermobility and clinically significant Beighton scores amongst EDS cases, particularly amongst kEDS (39/39, 100%), spEDS (24/25, 96.0%), and hEDS (153/160, 95.6%) subtypes. The most common musculoskeletal complications were decreased bone density (39/43, 90.7), joint pain (217/270, 80.4%), and hypotonia/weakness (79/140, 56.4%). Notably, joint pain was a significant feature observed in hEDS cases (161/170, 94.7%). Muscle/tendon rupture was almost entirely exclusive to vEDS cases, whilst sprains and subluxations/dislocations had a higher prevalence amongst hEDS, aEDS, spEDS, and cEDS cases. Abnormalities in the curvature of the spine were most notable in kEDS, spEDS, with scoliosis a prevalent feature amongst cEDS cases (45/59, 76.3%).

### Vascular complications

A high prevalence was noted of vascular complications such as cerebrovascular events (25/153, 16.3%), extracranial aneurysm (77/245, 31.4%), and arterial dissection/rupture (89/250, 35.5%) amongst vEDS cases. In particular, carotid cavernous fistulae were exclusively noted within vEDS patients. Cardiovascular complications such as early onset varicose veins, valvular incompetence, arterial tortuosity, fistulae, murmurs, and bundle-branch blocks were noted as prominent features amongst almost all subtypes, particularly cEDS, hEDS, and kEDS. Interestingly, cardiovascular dysautonomia was a major feature amongst most hEDS cases (56/63, 88.9%).

### Miscellaneous complications

Overall, chronic pain disproportionately affected the hEDS subtype (139/157, 88.5%) compared to other subtypes. Hypermobile EDS cases commonly exhibited chronic fatigue (61/63, 96.8%), gastrointestinal complications (57/63, 90.5%), and urological complications (59/159, 37.1%). Additional complications such as pneumothorax/hemothorax and bowel perforation were almost entirely exclusive to the vEDS subtype. Within this group, the mean age for first pneumothorax/hemothorax was 25.6 ± 9.0 years, whilst for bowel perforation it was 26.6 ± 10.9 years. Neuropsychiatric complications were noted in all kEDS, mEDS, and pEDS cases (100.0%), however, a substantial number of cases were also noted amongst vEDS and cEDS subtypes.

## Discussion

### Facial features

Craniofacial abnormalities were noted in almost all spEDS and aEDS cases. The gene variants noted in spEDS (*B4GALT7*/*B3GALT6*) cause significant impairment to glycosaminoglycan biosynthesis, producing a perturbed deposition of type I collagen, a disorganized ECM, and a misshapen and partly absent cartilage structure within connective tissue (11). Furthermore, during early development these variants can result in absent ossified bone structure, resulting in the clinical appearance of a short, round face with midface hypoplasia ± frontal bossing and proptosis (11). For aEDS specific variants in EXON6 *COL1A1* or *COL1A2* prevent cleavage of the collagen type I N-propeptide and disrupt the normal interhelical cross-linking of collagen fibers, which can cause significant bone and soft tissue abnormalities (11). Interestingly, similar craniofacial abnormalities are seen in osteogenesis imperfecta, which is also caused by variants in *COL1A1* or *COL1A2* and can show clinical overlap (12).

We noted that all BCS cases, and the majority of kEDS cases presented with blue/gray sclera and a thin cornea. BCS is due to biallelic variants in *ZNF469* or *PRDM5*. These genes encode proteins that are crucial role in the normal development of the anterior chamber segment and cornea, and maintenance of the ECM (13). Thus, pathogenic variants result in downstream thinning of the sclera, producing a bluish appearance. Both sensorineural and conductive deafness are seen in BCS, in addition to hypermobile tympanic membranes which can result in devastating polysensory loss when coupled with ophthalmic complications (7, 13).

### Musculoskeletal complications

Joint hypermobility and elevated Beighton scores were associated with most EDS subtypes, with many EDS subtypes showing some degree of joint laxity in their clinical presentation (2). It was also prevalent in rarer subtypes such as kEDS and spEDS, as well as hEDS. In *PLOD1*-related kEDS, the deficiency in collagen lysyl hydroxylase 1 causes under hydroxylation of lysyl residues in collagens and impairs the cross-link formation with consequent mechanical instability of joint ligaments (14). Similarly, as previously mentioned, the gene variants noted in spEDS (*B4GALT7*/*B3GALT6*) cause perturbed deposition of type I collagen, a disorganized ECM within connective tissues, contributing to joint hypermobility (11).

Though a consistent genetic association has not yet been discovered for hEDS, transcriptome profiling of hEDS fibroblasts has shown a differential expression of many adhesion molecule-encoding genes including members of cadherins, protocadherins and desmosomes, which are involved in the formation of specialized cell-cell junction complexes essential for maintaining epithelial integrity, morphogenesis, and tissue architecture (15). It is hypothesized that these transcriptional changes that suggest a fibroblast-to-myofibroblast transition, may cause deficiencies in the cytoskeletal architecture needed for joint stability (16). This could explain the high prevalence of sprains and subluxation/dislocations observed amongst hEDS, aEDS, spEDS, and cEDS cases. Furthermore, it is also these ECM/cytoskeletal deficiencies that contribute to weak spinal muscles, resulting in a lack of spinal column support and manifesting with spinal deformities as noted in kEDS, spEDS and cEDS cases (17).

Interestingly, we noted osteopenia/osteoporosis as a common complication amongst all EDS subtypes. Recent research has shown the various roles of collagens in osteoblastogenesis within the ECM (18, 19). An estimated 90% of the ECM in bone is comprised of collagen [primarily type I collagen, with lesser amounts of type V (∼10%)], which serves as a tissue scaffold but also provides a substrate for cell anchorage and regulates bioavailability of growth factors and cytokines (19). It is the collagen fibrils, containing type I and V collagen molecules, which are cross-linked in bone osteogenesis. Therefore, as expected variants causing altered or decreased collagen I and V (cEDS, vEDS, aEDS, and cvEDS), cause impaired bone mineralization (20). In addition, type III collagen is expressed at high levels during embryonic skeletal development and is expressed by osteoblasts for trabecular bone development. Thus, loss of function variants in *COL3A1* (vEDS) can impair osteoblast differentiation (19). Consequently, bone mineralization was substantiated in the clinical setting, where EDS patients (cEDS/hEDS) had reduced bone mineral density and bone quality, with an increased prevalence of vertebral fractures, compared to matched control subjects (21).

Joint pain is common in EDS patients (80.4%), particularly within hEDS cases (94.7%). Consequently, joint pain, particularly hypermobility-related pain is one of the leading disability determinants in joint hypermobility related disorders (22). The natural history of pain in joint hypermobility and related disorders indicates that pain is usually nociceptive (physical pain caused by structural dysfunction) at the beginning of the disease (23). Furthermore, studies have suggested that patients with EDS may experience neuropathic pain, whilst others suggest joint pain spreads beyond the joints, causing fatigue, and resembling fibromyalgia (24, 25). In contrast, a pilot study of 24 EDS patients (cEDS, vEDS, hEDS) demonstrated the presence of small fiber neuropathy, supporting the additional features of paresthesias, dysesthesias, neuropathic pain, and autonomic symptoms, which are often observed in EDS patients (26).

### Vascular complications

These are especially common in vEDS (variants in *COL3A1*/*COL1A1*) and are the most significant due to the risk of life-threatening features of tissue fragility that can cause cerebrovascular events, aneurysm, and arterial dissection/rupture (27). Vascular EDS patients with pathogenic variants in *COL3A1*, have structural defects of the C-propeptide chain of collagen type III, defects that are characterized by decreased thermal stability and abnormalities in proteolytic processing that can produce procollagen suicide (28). Substitutions of triple helical glycine residues and splice donor site variants, leading to exon skipping, are generally associated with a shorter life expectancy, whereas variants leading to *COL3A1* haploinsufficiency are usually associated with a milder phenotype, a delay in the onset of complications, and a longer life expectancy (29, 30). Interestingly, we noted 3 cases of vEDS autosomal recessive inheritance, with one case being compound heterozygous for two *COL3A1* variants, and two cases being homozygous for a *COL3A1* missense variant, with all parents unaffected (31). For vEDS patients, there are also significant medical challenges in early adulthood, with American and European clinical data suggesting ∼20–25% of vEDS patients will have a major vascular complication by age 20, and ∼70–80% having at least one major complication by age 40 (29, 32). Furthermore, early vascular complications or bowel rupture are well-recognized even before adolescence (31). Given these alarming statistics, early diagnosis is therefore vital. The most common presentation in childhood is easy bruising that may be accompanied by skin lucency and vascular visibility, as well as a possible family history of vascular complications (33).

Amongst all subtypes, we noted a prevalence of cardiovascular complications. This is an important finding, as non-vascular EDS subtypes can still present with vascular complications. It has been noted that vascular complications are most frequently reported in mcEDS (*CHST14/DSE*) and in clEDS (*TNXB*), occur in two thirds of the patients, respectively (30). Furthermore, cEDS, cvEDS, dEDS, and kEDS subtypes also show a lower, but nonetheless important risk of vascular complications, such as intracranial hemorrhages, arterial aneurysms, and arterial dissections, compared to vEDS (30).

We also observed a high prevalence of cardiovascular dysautonomia amongst hEDS cases. There is growing recognition of the association between hEDS and cardiovascular autonomic dysfunction, which can present as orthostatic intolerance, orthostatic hypotension, postural orthostatic tachycardia syndrome, or neurally mediated hypotension (34). It was noted in a cohort study that ∼50% of adults with hEDS or hypermobility spectrum disorder had diagnostic features of cardiovascular dysautonomia (35). So far, no convincing plausible mechanism has been identified, although there are multiple hypotheses such as abnormal connective tissue in dependent blood vessels causing abnormal vein distention (36), neuropathy with tissue laxity and vasoactive mediation (37), and pathogenic adrenergic and muscarinic autoantibody development (34).

### Miscellaneous complications

The most notable group presenting with additional complications were from hEDS cases, with high rates of chronic pain, chronic fatigue, gastrointestinal complications (constipation, diarrhea, irritable bowel syndrome, reflux and/or hepatitis), and urological complications (urinary incontinence, bladder cysts/rupture and/or uterine rupture). As previously mentioned, multiple hypotheses have been proposed to try and explain the multi-system involvement of hEDS patients. For hEDS patients, a reduced expression of specific fibroblast miRNA has been demonstrated, which is implicated in the Wnt pathway for normal ECM regulation. Decreased levels of this miRNA were also found in cerebrospinal fluid and serum of patients with fibromyalgia (38), implying a possible involvement of specific miRNA signatures or a common disease pattern in both conditions (16). In addition, clinical studies have shown that hEDS patients suffer from widespread pain and manifest signs compatible with central sensitization, causing fibromyalgia-like, widespread pain and hyperalgesia to painful stimuli (39). This suggests that the chronic pain and fatigue experienced may reflect abnormal central processing of the nociceptive input in the dorsal horn neurons, possibly involving the descending modulatory system (40, 41). These pathways most likely input into the gastrointestinal (42) and urological systems (43), further explaining the multi-system presentation.

Spontaneous pneumothoraces/hemothoraces was noted amongst vEDS cases. Type III collagen in the lung is expressed in vessels, parenchymal fibroblasts and is particularly rich in the pleural linings (44). Thus, pulmonary complications of vEDS are proposed to result from poor tissue integrity (45). In addition, there may be longstanding damage from inefficient repair of the lung parenchyma and pulmonary vessels after injury, with type I collagen favored over the absent type III collagen (46). We also noted that for vEDS cases, age of first pneumothorax/hemothorax was 25.6 ± 9.0 years. Thus, spontaneous pneumothorax/hemothorax is an early manifestation of vEDS and frequently precedes an arterial complication or intestinal perforation (first presentation at 26.6 ± 10.9 years) (47).

Neuropsychiatric complications were prevalent amongst many of the EDS subtypes, possibly indicating the significant impact that joint hypermobility and EDS complications have on the quality of life of EDS patients. Anxiety symptoms and disorders have a well-established link with hypermobility, yet growing evidence also now points toward associations across psychiatric diagnoses, notably with affective illnesses, increasingly with neurodevelopmental disorders and with stress-sensitive medical conditions, including fibromyalgia, chronic fatigue syndrome, and irritable bowel syndrome (48). However, future research is needed to investigate the genetic, neural and psychophysiological mechanisms for such mind-body interactions.

### Limitations

Due to the retrospective nature of this study, severity of clinical features could not be assessed. Furthermore, due to a lack of a standardized assessment of craniofacial characteristics and cerebrovascular, cardiovascular, and neuropsychiatric complications, a sub-analysis of individual features could not be undertaken. It must also be noted that in many cases (particularly vEDS cases) that it was not possible to gather information on the scans that were used to identify aneurysms and/or dissection and whether the patient presented symptomatically, or if this was an incidental finding. Given that assessments were performed by physicians and EDS clinicians there is an inherent subjectivity when performing Beighton score assessments. The systematic and predefined search strategy aimed to identify all potentially relevant studies, but the risk of selection bias is not negligible. Due to the rarity of some EDS subtypes, only small case numbers (predominantly from family pedigrees) were reported, which may impact the generalizability of the findings.

In conclusion, the extracutaneous features of the EDS subtypes, as well as the associated complications of each EDS subtype have been presented. We observed significant features that were specific to EDS subtypes, which can help the clinician in an accurate individual assessment of EDS and assist in the decision to undertaken next generation sequencing technologies for a molecular diagnosis. Furthermore, having a comprehensive understanding of the possible complications (particularly for vEDS) may help diagnose and treat EDS prior to the development of substantial comorbidities and/or additional complications. To expand our understanding of EDS and to help optimize management and care guidelines, future research should be focused on further systematic documentation of clinical features and complications, as well as the natural history of each EDS subtype.

## Data availability statement

Datasets from this systematic review can be provided, upon reasonable request by contacting the corresponding author.

## Author contributions

BD collected preliminary data, contributed to the analysis, and development of the original and revised manuscripts. ML, IH, FM, SS, and IW contributed to the analysis of included manuscript for this review and significant contributions to the revision of the manuscript. JS contributed to the statistical analysis of the raw data, collation of tables, and revision of the manuscript. NB was supervisor of this project who contributed to the design and analysis of this project, review of all manuscript included in the review, and significant contribution to the original and revised manuscripts. All authors contributed to the article and approved the submitted version.
