# Supplementary material for: Extracutaneous features and complications of the Ehlers-Danlos syndromes: A systematic review
Source: Front Med (Lausanne). 2023 Jan 23;10:1053466. doi: 10.3389/fmed.2023.1053466 (PMC9899794; doi:10.3389/fmed.2023.1053466)
Supplement: Supplementary file 1 [file Data_Sheet_1.docx]

***Dermatologic manifestations and diagnostic assessments of the Ehlers-Danlos syndromes: A systematic review***

Doolan BJ, Lavallee M, Labine B, Hausser I, Pope FM, Seneviratne SL, Winship IM, Burrows NP

_________________________________________________________________________________

***Supplementary Information:***

**Figure S1:** PRISMA flow diagram for EDS systematic review

***
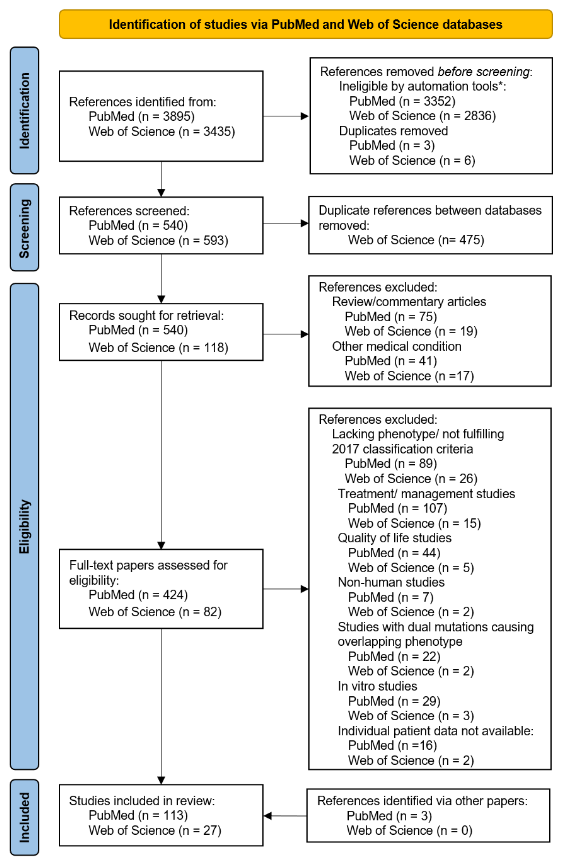
***

*Automation tools: only published articles written in English, between 01 March 2017 – 12

January 2022 were included. Review articles and editorials were excluded from the search.

**Table S1:** The 2017 international Ehlers–Danlos syndrome classification. Adapted from Malfait et al., 2017.

| **EDS type*** | **Genetic basis** | **Inheritance** | **Protein** | **Major clinical criteria** | **Minor clinical criteria** | **Minimal criteria required** |
| --- | --- | --- | --- | --- | --- | --- |
| Classical EDS | *COL5A1 COL5A2 COL1A1* | AD | Type V collagen Type I collagen | 1.Skin hyperextensibility and atrophic scarring 2.Generalized joint hypermobility(GJH) | 1.Easy bruising 2.Soft, doughy skin 3.Skin fragility (or traumatic splitting) 4.Molluscoid pseudotumours 5.Subcutaneous spheroids 6.Hernia (or history thereof) 7.Epicanthal folds 8.Complications of joint hyper-mobility (e.g., sprains, luxation/subluxation, pain, flexible flatfoot) 9.Family history of a first degree relative who meets clinical criteria | (1): skin hyperextensibility and atrophic scarring Plus Either major criterion (2): GJH– and/or: at least three minor criteria. Confirmatory molecular testing is obligatory to reach a final diagnosis. |
| Classical-like EDS | *TNXB* | AR | Tenascin XB | 1.Skin hyperextensibility, with velvety skin texture and absence of atrophic scarring 2.Generalised joint hypermobility with or without recurrent dislocations (most commonly shoulder and ankle) 3.Easy bruisable skin/spontaneous ecchymoses | 1. Foot deformities: broad/plump fore-foot, brachydactyly with excessive skin; pes planus; hallux valgus; piezogenic papules 2.Edema in the legs in absence of cardiac failure 3.Mild proximal and distal muscle weakness 4.Axonal polyneuropathy 5.Atrophy of muscles in hands and feet 6.Acrogeric hands, mallet finger(s), clinodactyly, brachydactyly 7.Vaginal/uterus/rectal prolapse | All three major criteria AND a family history compatible with autosomal recessive transmission. Confirmatory molecular testing is obligatory to reach a final diagnosis |
| Vascular EDS | *COL3A1 COL1A1* | AD | Type III collagen Type I collagen | 1. Family history of vEDS with documented causative variant in COL3A1 2.Arterial rupture at a young age 3.Spontaneous sigmoid colon perforation in the absence of known diverticular disease or other bowel pathology 4.Uterine rupture during the third trimester in the absence of previous C-section and/or severe peripartum perineum tears 5.Carotid-cavernous sinus fistula (CCSF)formation in the absence of trauma | 1.Bruising unrelated to identified trauma and/or in unusual sites such as cheeks and back 2.Thin, translucent skin with increased venous visibility 3.Characteristic facial appearance 4.Spontaneous pneumothorax 5.Acrogeria 6.Talipes equinovarus 7.Congenital hip dislocation 8.Hypermobility of small joints 9.Tendon and muscle rupture 10.Keratoconus 11.Gingival recession and gingival fragility 12.Early onset varicose veins (under age 30 and nulliparous if female) | A family history of the disorder, arterial rupture or dissection in individuals less than 40 years of age, unexplained sigmoid colon rupture, or spontaneous pneumothorax in the presence of other features consistent with vEDS should all lead to diagnostic studies to determine if the individual has vEDS. Confirmatory molecular testing is obligatory to reach a final diagnosis. |
| Hypermobile EDS | Unknown | AD | Unknown | Criteria 1: Beighton score of • ≥6 pre-pubertal children and adolescents • ≥5 pubertal men and woman to age 50 • ≥4 men and women over the age of 50 Criterion 2: (Two or more of the following features (A, B, or C) must be present) **A**: (Five must be present) • Unusually soft or velvety skin • Mild skin hyperextensibility • Unexplained striae distensae or rubae at the back, groins, thighs, breasts and/or abdomen in adolescents, men or pre-pubertal women without a history of significant gain or loss of body fat or weight • Bilateral piezogenic papules of the heel • Recurrent or multiple abdominal hernia(s) • Atrophic scarring involving at least two sites and without the formation of truly papyraceous and/or hemosideric scars as seen in classical EDS • Pelvic floor, rectal, and/or uterine prolapse in children, men or nulliparous women without a history of morbid obesity or other known predisposing medical condition • Dental crowding and high or narrow palate • Arachnodactyly, as defined in one or more of the following: (i) positive wrist sign (Walker sign) on both sides, (ii) positive thumb sign (Steinberg sign) on both sides • Arm span-to-height ratio ≥1.05 • Mitral valve prolapse (MVP) mild or greater based on strict echocardiographic criteria • Aortic root dilatation with Z-score >+2 **B**: Positive family history; one or more first-degree relatives independently meeting the current criteria for hEDS **C**: (≥ 1 feature must be present) • Musculoskeletal pain in two or more limbs, recurring daily for at least 3 months • Chronic, widespread pain for ≥3 months • Recurrent joint dislocations or frank joint instability, in the absence of trauma | - | A clinical diagnosis of hEDS is made if all 3 criteria are met. Criteria 3 specifically excludes the diagnosis if features of other types of EDS/connective tissue disorders are suspected and/or adjusts requirements of Criterion 2 in the presence of acquired connective tissue disorder |
| Arthrochalasia EDS | *COL1A1 COL1A2* | AD | Type I collagen | 1.Congenital bilateral hip dislocation 2.Severe GJH, with multiple dislocations/subluxations 3.Skin hyperextensibility | 1.Muscle hypotonia 2.Kyphoscoliosis 3.Radiologically mild osteopenia 4.Tissue fragility, including atrophic scars 5.Easy bruisable skin | Congenital bilateral hip dislocation Plus– Either major criterion (3): skin hyperextensibility– Or major criterion (2): severe GJH with multiple dislocations/subluxations and at least two other minor criteria. Confirmatory molecular testing is obligatory to reach a final diagnosis |
| Dermatosparaxis EDS | *ADAMTS2* | AR | ADAMTS-2 | 1.Extreme skin fragility with congenital or postnatal skin tears 2.Characteristic craniofacial features, which are evident at birth or early infancy, or evolve later in childhood 3.Redundant, almost lax skin, with excessive skin folds at the wrists and ankles 4.Increased palmar wrinkling 5.Severe bruisability with a risk of subcutaneous hematomas and haemorrhage 6.Umbilical hernia 7.Postnatal growth retardation 8.Short limbs, hand and feet 9.Perinatal complications due to connective tissue fragility | 1.Soft and doughy skin texture 2.Skin hyperextensibility 3.Atrophic scars 4.GJH 5.Complications of visceral fragility (e.g., bladder rupture, diaphragmatic rupture, rectal prolapse) 6.Delayed motor development 7.Osteopenia | (1): Extreme skin fragility AND major criterion (2): characteristic craniofacial features Plus Either: one other major criterion And/or: three minor criteria Confirmatory molecular testing is obligatory to reach a final diagnosis. |
| Kyphoscoliotic EDS | *PLOD1 FKBP14* | AR | LH1 FKBP22 | 1.Congenital muscle hypotonia 2.Congenital or early onset kypho-scoliosis (progressive or non-progressive) 3.GJH with dislocations/ subluxations (shoulders, hips, and knees in particular) | 1.Skin hyperextensibility 2.Easy bruisable skin 3.Rupture/aneurysm of a medium-sized artery 4.Osteopenia/osteoporosis 5.Blue sclerae 6.Hernia (umbilical or inguinal) 7.Pectus deformity 8.Marfanoid habitus 9.Talipes equinovarus 10.Refractive errors (myopia, hypermetropia) | (1): congenital muscle hypotonia AND major criterion (2): congenital or early-onset kyphoscoliosis Plus Either major criterion (3): GJH And/or three minor criteria(either general or gene-specific criteria). Confirmatory molecular testing is obligatory to reach a final diagnosis. |
| Brittle Cornea Syndrome | *ZNF469 PRDM5* | AR | ZNF469 PRDM5 | 1.Thin cornea, with or without rupture (central corneal thickness often<400mm) 2.Early onset progressive keratoconus 3.Early onset progressive keratoglobus 4.Blue sclerae | 1.Enucleation or corneal scarring as a result of previous rupture 2.Progressive loss of corneal stromal depth, especially in central cornea 3.High myopia, with normal or moderately increased axial length 4.Retinal detachment 5.Deafness, often with mixed conductive and sensorineural components, progressive, higher frequencies of-ten more severely affected 6.Hypercompliant tympanic membranes 7.Developmental dysplasia of the hip 8.Hypotonia in infancy, usually mild if present 9.Scoliosis 10.Arachnodactyly 11.Hypermobility of distal joints 12.Pes planus, hallux valgus 13.Mild contractures of fingers (especially 5th) 14.Soft, velvety skin, translucent skin | (1): thin cornea, with or without rupture (central corneal thickness often <100micrometer) Plus Either: at least one other major criterion and/or three other minor criteria. Confirmatory molecular testing is obligatory to reach a final diagnosis. |
| Spondylodysplastic EDS | *B4GALT7 B3GALT6 SLC39A13* | AR | β4GalT7 β3GalT6 ZIP13 | 1.Short stature (progressive in childhood) 2.Muscle hypotonia (ranging from severe congenital, to mild later-onset) 3.Bowing of limbs | 1.Skin hyperextensibility, soft, doughy skin, thin translucent skin 2.Pes planus 3.Delayed motor development 4.Osteopenia 5.Delayed cognitive development PLUS individual minor criteria based on mutation | (1): short stature AND major criterion (2): muscle hypotonia Plus Characteristic radiographic abnormalities and at least three other minor criteria (general or type-specific). Confirmatory molecular testing is obligatory to reach a final diagnosis. |
| Musculocontractural EDS | *CHST14 DSE* | AR | D4ST1 DSE | 1.Congenital multiple contractures, characteristically adduction-flexion contractures and/or talipes equinovarus (clubfoot) 2.Characteristic craniofacial features, which are evident at birth or in early infancy 3.Characteristic cutaneous features including skin hyperextensibility, easy bruisability, skin fragility with atrophic scars, increased palmar wrinkling | 1.Recurrent/chronic dislocations 2.Pectus deformities (flat, excavated) 3.Spinal deformities (scoliosis, kyphoscoliosis) 4.Peculiar fingers (tapering, slender, cylindrical) 5.Progressive talipes deformities (valgus, planus, cavum) 6.Large subcutaneous hematomas 7.Chronic constipation 8.Colonic diverticula 9.Pneumothorax/pneumohemothorax 10.Nephrolithiasis/cystolithiasis 11.Hydronephrosis 12.Cryptorchidism in males 13.Strabismus 14.Refractive errors(myopia, astigmatism) 15.Glaucoma/elevated intraocular pressure | At birth or in early childhood: Major criterion (1): Congenital multiple contractures AND (2) characteristic craniofacial features In adolescence and in adulthood: Major criterion (1): Congenital multiple contractures AND (3) characteristic cutaneous features. Confirmatory molecular testing is obligatory to reach a final diagnosis. |
| Myopathic EDS | *COL12A1* | AR AD | Type XII collagen | 1.Congenital muscle hypotonia, and/or muscle atrophy, that improves with age 2.Proximal joint contractures (knee, hip, and elbow) 3.Hypermobility of distal joints | 1.Soft, doughy skin 2.Atrophic scarring | (1): congenital muscle hypotonia that improves with age Plus Either: one other major criterion And/or: three minor criteria. Confirmatory molecular testing is obligatory to reach a final diagnosis. |
| Periodontal EDS | *C1R C1S* | AD | C1r C1s | 1.Severe and intractable periodontitis of early onset (childhood or adolescence) 2.Lack of attached gingiva 3.Pretibial plaques 4.Family history of a first-degree relative who meets clinical criteria | 1.Easy bruising 2.Joint hypermobility, mostly distal joints 3.Skin hyperextensibility and fragility, abnormal scarring (wide or atrophic) 4.Increased rate of infections 5.Hernias 6.Marfanoid facial features 7.Acrogeria 8.Prominent vasculature | (1): severe and intractable periodontitis of early onset (childhood or adolescence) OR major criterion (2): lack of attached gingiva Plus At least two other major criteria and one minor criterion. Confirmatory molecular testing is obligatory to reach a final diagnosis |
| Cardiac Valvular EDS | *COL1A2* | AR | Type I collagen | 1.Severe progressive cardiac-valvular problems (aortic valve, mitral valve); 2.Skin involvement: skin hyperextensibility, atrophic scars, thin skin, easy bruising; and. 3.Joint hypermobility (generalized or restricted to small joints). | 1.Inguinal hernia 2.Pectus deformity (especially pectus excavatum) 3. Joint dislocations 4. Foot deformities: pes planus, pes planovalgus, hallux valgus | (1) first major criterion plus a family history compatible with autosomal recessive transmission, and either one other major criterion or at least two minor criteria. Confirmatory molecular testing is obligatory to reach a final diagnosis. |
| AR = Autosomal recessive, AD = Autosomal dominant, GJH = Generalised joint hypermobility  * Note that classical-like type 2 Ehlers-Danlos Syndrome (clEDS2) has been proposed as 14^th^ subtype, which is characterised by generalised joint hypermobility with recurrent joint dislocations, redundant and hyperextensible skin with poor wound healing and abnormal scarring, easy bruising, and osteopenia/osteoporosis. | | | | |  |  |

**Table S2:** Overview of search strings used to find relevant references in the electronic databases of PubMed and Web of Science.

| **EDS Subtype** | **Search string PubMed** | **Search string Web of Science** |
| --- | --- | --- |
| Overview of all EDS cases | (ehlers-danlos [Title/Abstract]) NOT (Review[Publication Type]) | (TI=(ehlers-danlos syndrome)) AND AB=(Ehlers-Danlos Syndrome) |
| *Disorders of collagen primary structure and collagen processing* | |  |
| Classical EDS (COL5A1/2) | ("Ehlers-Danlos syndrome"[Title/Abstract] AND (classic*[Title/Abstract] OR COL5A1[Title/Abstract] OR COL5A2[Title/Abstract])) OR "Ehlers-Danlos syndrome type I"[Title/Abstract] | TS=(("Ehlers-Danlos syndrome" AND (classic* OR COL5A1 OR COL5A2)) |
| Vascular EDS | ("Ehlers-Danlos syndrome"[Title/Abstract] AND (vascular*[Title/Abstract] OR COL3A1[Title/Abstract] OR COL1A1[Title/Abstract])) | TS=((“Ehlers-Danlos syndrome" AND (vascular OR COL3A1 OR COL1A1) |
| Arthochalasia | ("Ehlers-Danlos syndrome"[Title/Abstract] AND arthrochalasia[Title/Abstract]) OR "Ehlers-Danlos syndrome type VIIA”[Title/Abstract] | TS=((“Ehlers-Danlos syndrome" AND arthrochalasia) |
| Dermatosparaxis | ("Ehlers-Danlos syndrome"[Title/Abstract] AND (dermatospara*[Title/Abstract] OR ADAMTS2[Title/Abstract])) | TS=((“Ehlers-Danlos syndrome" AND (dermatospara* OR ADAMTS2)) |
| Cardiac Valvular + Classical EDS | Ehlers-Danlos syndrome[Title/Abstract] AND ((COL1A1[Title/Abstract]) OR COL1A2[Title/Abstract])) | TS=(“Ehlers-Danlos syndrome" AND (COL1A1 OR COL1A2)) |
| *Disorders of collagen folding and collagen crosslinking* | |  |
| Kyphoscoliotic EDS | ("Ehlers-Danlos syndrome"[Title/Abstract] AND (kyphoscolio*[Title/Abstract] OR PLOD1[Title/Abstract])) ("Ehlers-Danlos syndrome"[Title/Abstract] AND (FKBP14[Title/Abstract]) | TS=((“Ehlers-Danlos syndrome" AND (kyphoscolio* OR PLOD1)) TS=(“Ehlers-Danlos syndrome" AND FKBP14) |
| Classical-like EDS | Ehlers-Danlos syndrome[Title/Abstract] AND (TNXB[Title/Abstract] OR “tenascin-x”[Title/Abstract]) | TS=(“Ehlers-Danlos syndrome" AND (TNXB OR “tenascin-x”)) |
| *Disorders of structure and function of the myomatrix* | |  |
| Classical-like EDS | Ehlers-Danlos syndrome[Title/Abstract] AND (TNXB[Title/Abstract] OR “tenascin-x”[Title/Abstract]) | TS=(“Ehlers-Danlos syndrome" AND (TNXB OR “tenascin-x”)) |
| Myopathic EDS | Ehlers-Danlos syndrome[Title/Abstract] AND COL12A1[Title/Abstract] | TS=("Ehlers-Danlos syndrome" AND COL12A1) |
| *Disorders of glycosaminoglycan biosynthesis* | |  |
| Musculocontractural EDS | ("Ehlers-Danlos syndrome"[Title/Abstract] AND (musculocontractural[Title/Abstract] OR CHST14[Title/Abstract] OR DSE[Title/Abstract])) OR "Ehlers-Danlos syndrome type VIB"[Title/Abstract] OR "adducted Thumb-clubfoot syndrome"[Title/Abstract] OR “Ehlers-Danlos syndrome Kosho type”[Title/Abstract] OR “D4ST1-deficient Ehlers-Danlos syndrome”[Title/Abstract] | TS=((“Ehlers-Danlos syndrome" AND (musculocontractural OR CHST14 OR DSE)) OR “Ehlers-Danlos syndrome type VIB” OR “adducted thumb-clubfoot syndrome” OR “Ehlers-Danlos syndrome Kosho type” OR “D4ST1-deficient Ehlers-Danlos syndrome”) |
| Spondylo-dysplastic EDS | "Ehlers-Danlos syndrome"[Title/Abstract] AND (B4GALT7[Title/Abstract]) "Ehlers-Danlos syndrome"[Title/Abstract] AND (B3GALT6[Title/Abstract]) "Ehlers-Danlos syndrome"[Title/Abstract] AND (SLC39A13[Title/Abstract]) | TS=(“Ehlers-Danlos syndrome” AND (B4GALT7)) TS=(“Ehlers-Danlos syndrome” AND (B3GALT6)) TS=(“Ehlers-Danlos syndrome" AND (SLC39A13)) |
| *Disorders of intracellular processes* |  |  |
| Brittle Cornea Syndrome | “brittle cornea syndrome"[Title/Abstract] AND (ZNF469[Title/Abstract] OR PRDM5[Title/Abstract]) | TS=("brittle cornea syndrome" AND (ZNF469 OR PRDM5)) |
| *Disorders of complement pathway* |  |  |
| Periodontal EDS | "Ehlers-Danlos syndrome"[Title/Abstract] AND (periodontal[Title/Abstract] OR C1R[Title/Abstract] OR C1S[Title/Abstract]) | TS=(“Ehlers-Danlos syndrome” AND (periodontal OR C1R OR C1S)) |
| Hypermobile EDS | "Ehlers-Danlos syndrome"[Title/Abstract] AND hypermobile[Title/Abstract] | TS=((“Ehlers-Danlos syndrome" AND hypermobile) |
| Classical-like Type 2 EDS | "Classical-like type 2"[Title/Abstract] AND "AEBP1"[Title/Abstract] | TS=((“Ehlers-Danlos syndrome" AND arthrochalasia) |
